# Oropharyngeal CSF Leak Secondary to Anterior Cervical Discectomy and Fusion

**DOI:** 10.1155/2018/7629184

**Published:** 2018-07-31

**Authors:** Wyatt J. Weinheimer, Justin G. Rowley, Randal Otto, John Floyd, Philip G. Chen

**Affiliations:** ^1^Department of Otolaryngology Head Neck Surgery, University of Texas Health at San Antonio, 7703 Floyd Curl Dr, MC 7777, San Antonio, TX 78229, USA; ^2^UT Long School of Medicine, University of Texas, Austin, TX 78712, USA; ^3^Department of Neurosurgery, University of Texas Health at San Antonio, 7703 Floyd Curl Drive, MC 7843, San Antonio, TX 78229, USA

## Abstract

We present a case of an oropharyngeal cerebrospinal fluid (CSF) fistula in a patient that presented with headache, rhinorrhea, and pneumocephalus years after an anterior cervical discectomy and fusion. Imaging suggested a defect in the fovea ethmoidalis, but endoscopic surgery revealed the defect in the oropharynx. A second procedure was performed to remove the spinal hardware and repair the leak. This case is not only unique in the literature but also highlights the importance of maintaining a broad differential diagnosis to include rare complications and shows that despite dramatic improvements in imaging, locating CSF leaks still presents a challenge.

## 1. Introduction

Cerebrospinal fluid (CSF) leaks represent a pathological connection and communication between the subarachnoid space with another nonnative cavity. CSF leakage can result from innumerable processes: iatrogenic, trauma, tumors, or spontaneous disruption of a barrier. Patients often present when the CSF leaks are large enough to result in pneumocephalus or meningitis, a serious and life-threatening condition that can result in permanent neurologic deficits.

Identification of the site of the leak can pose a challenge. Multiple imaging modalities including high-resolution computed tomography (HRCT), CT cisternography/myelography, and magnetic resonance imaging can be and have been used. All have been shown to be useful, but the sensitivity and specificity varies greatly depending on size, location, etiology, and status of leak (active versus inactive) [1–4].

Anterior cervical discectomy and fusion (ACDF) procedures are very common and relatively safe. Although rare, patients undergoing ACDF surgeries can develop intraoperative CSF leaks through accidental dural breach during the procedure (0.2% to 1.8% of cases) with the vast majority of these being noted and repaired at the time of surgery [5, 6]. Development of a postoperative fistula is even less common (<0.2%) and usually presents as clear drainage externally from the surgical neck incision [6].

The purpose of this study is to discuss the workup and management of a patient that presented five years after ACDF with chronic dysphagia, acute headache, pneumocephalus, and signs of meningitis that was subsequently found to have a CSF-oropharyngeal fistula. Imaging suggested that the site of the CSF communication was the left fovea ethmoidalis. The correct anatomic location of the fistula, however, was not identified until a negative endoscopic exploration of the skull base led to identifying the oropharyngeal defect.

After a thorough literature review, we identified 3 case reports of post-ACFD fistula presenting as a neck mass and dysphagia [7–9] and one case of spinal hardware eroding into the esophagus and two cases of erosion into the pharynx [10–12]. To the best of our knowledge, there have been no cases reported of oropharyngeal mucosal erosion from ACDF hardware with resultant delayed CSF fistula.

## 2. Case Report

A 64-year-old male presented to the emergency room with a 2-week history of a severe, persistent headache. The patient's pertinent past medical and surgical history included obesity and an anterior cervical discectomy and fusion five years prior. A CT scan of the head followed by an MRI of the brain and spine revealed extensive pneumocephalus and concerns for meningitis. The MRI of the spine showed the ACDF hardware but did not reveal surrounding defects. The neurosurgery team was consulted, and the patient was admitted.

**Figure 1:**
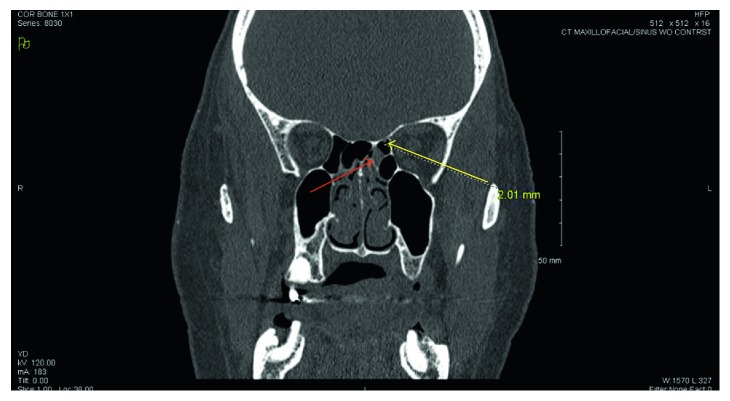
High-resolution CT showing area suspicious for a small skull base defect (yellow arrow) and adjacent opacification in the posterior ethmoid sinus (red arrow).

CT cisternogram/myelogram and high-resolution CT sinus were obtained. The cisternogram/myelogram was negative for leak at the skull base and cervical spine. CT imaging showed an air-fluid level within a left posterior ethmoid air cell with an apparent 2 mm adjacent osseous dehiscence along the fovea ethmoidalis, suspicious for the source of the CSF leak in this patient (Figure 1). The radiology report also commented that hardware from the anterior and posterior fusion between C3-C6 appeared intact without evidence of fistula or pseudomeningocele.

The otolaryngology/rhinology team was consulted due to the radiographic findings on the sinus CT. On further history and exam, the patient reported intermittent clear rhinorrhea and occasional salty tasting drainage. He denied any significant history of rhinosinusitis. Physical exam demonstrated an obese man who was uncomfortable. Holding the neck in flexion demonstrated clear fluid from the left nostril. The nasal endoscopy was normal. Based on clinical presentation and imaging, there was concern for a left skull defect.

The patient was taken to the operating room for identification and repair of the CSF leak. The neurosurgery team placed a lumbar drain and dilute fluorescein dye injected intrathecally. On nasal endoscopy, fluorescein dye was noted to be pooling in the nasopharynx throughout the case. A total sphenoethmoidectomy was performed, and the area of the potential defect was located and confirmed with intraoperative surgical navigation. Inspissated mucus was found at the area of mucosal thickening on CT (Figure 1) without evidence of fluorescein-dyed cerebrospinal fluid or bony dehiscence at the fovea ethmoidalis. The location was reconfirmed with surgical navigation. The right sinonasal cavity was evaluated without identification of any leak.

**Figure 2:**
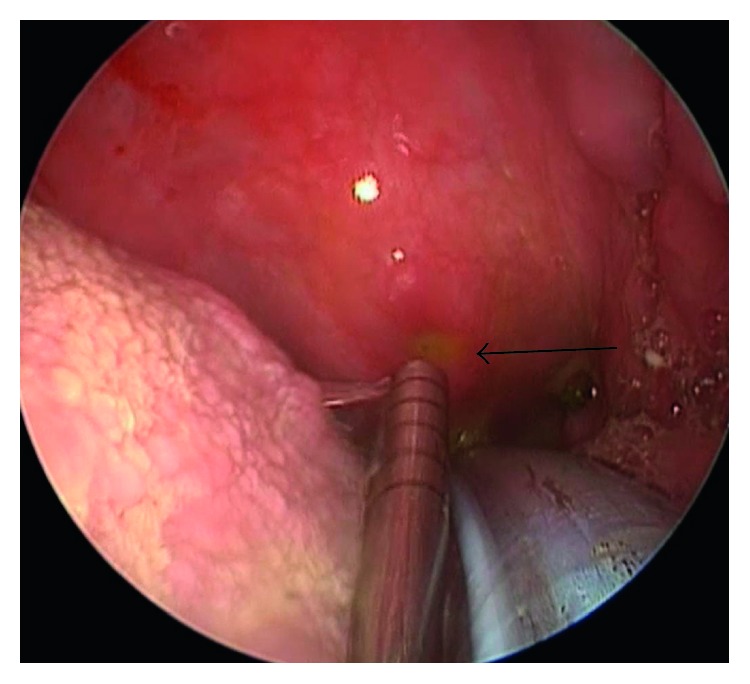
Endoscopic identification of fluorescein-dyed CSF in the oropharynx.

Subsequent endoscopic evaluation of the nasopharynx and oropharynx revealed significant pooling of fluorescein, but both openings to the eustachian tubes were normal. Close inspection of the mid-oropharynx, however, revealed a bulge in the posterior oropharynx, and a small, pinpoint fistula with actively leaking fluorescein-dyed CSF was found (Figure 2).

**Figure 3:**
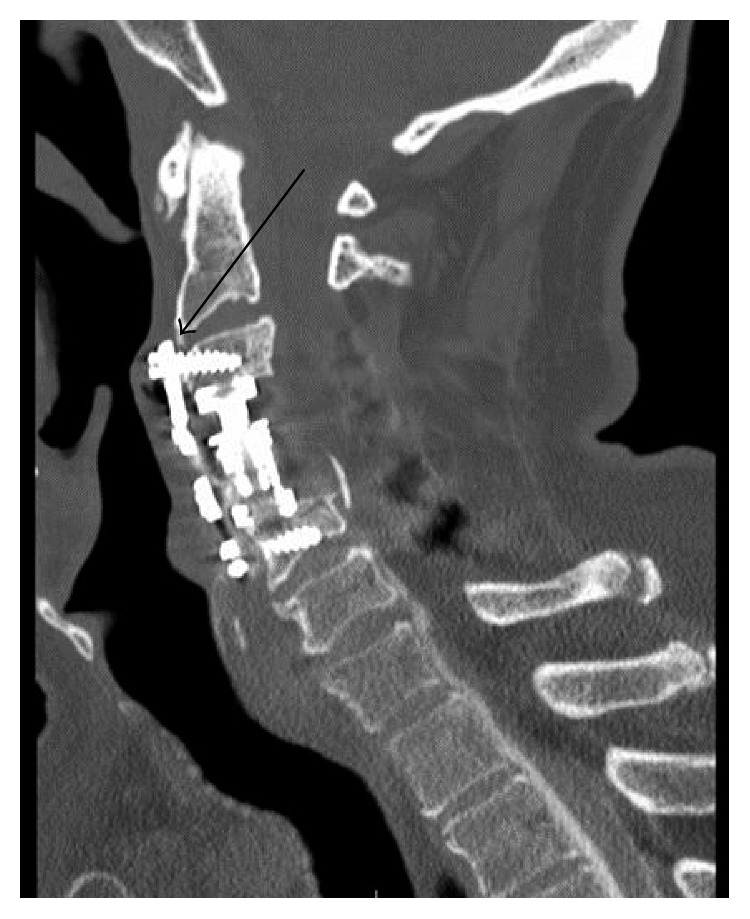
CT neck showing cervical hardware with thinning of the oropharyngeal mucosa. CT myelogram was negative for leak in this region.

The CT scan of the neck region was reviewed to evaluate the cervical hardware (Figure 3), and the head and neck surgery team was asked to assist neurosurgery with repair of the fistula. An anterior neck approach to the cervical hardware was performed. The anterior cervical hardware was removed and revealed a profuse CSF leak in association with the hardware. The defect was repaired with DuraSeal followed by rotation of the right omohyoid muscle into the site of the defect between the prevertebral fascia and the oropharyngeal mucosa. The neck incision was closed in a layered fashion. The patient was transported to the neurosurgery intensive care unit with the lumbar drain in place. He remained in the ICU on bed rest with the lumbar drain open at 10 ml/hr for 5 days. The patient was then allowed to mobilize, and the lumbar drain was removed. His headaches resolved, and repeat imaging showed resolution of the pneumocephalus. He was discharged and has done well with no evidence of CSF leak. A repeat CT myelogram of the spine at 8 months showed no signs of persistent leak.

## 3. Discussion

This case illustrates two points. The first is that CSF leak localization can be challenging [13, 14]. The second is that this is a first known case of delayed CSF leak after ACDF with an oropharyngeal fistula.

As observed in this case, localizing a CSF leak can be difficult. Multiple imaging modalities are used to localize defects. High-resolution CT scan has the benefit of wide available and noninvasive to the patient. It is useful for evaluating the sinonasal and bony anatomy [1–3]. A systematic review by Oakley et al. found sensitivity and specificity for identification of a CSF leak was between 44–100% and 45–100%, respectively, with most studies at the upper end of this range [2]. It is considered the first-line imaging modality for CSF leak localization.

MRI is another common option. CSF is bright on T2-weighted imaging and can be seen passing through a defect. While also noninvasive, it is more expensive than CT scanning. The sensitivity and specificity ranges from 56–94% and 57–100% [1–4].

CT cisternography/myelography combines intrathecal contrast injection followed by CT scanning to identify a fistula. Studies report relatively lower accuracy compared to other options [2]. This lower accuracy and the invasive nature make it less attractive as a first-line imagining modality.

Other options include MRI cisternography and single proton emission CT. Some studies suggest improved sensitivity and specificity with these modalities, but they are not widely available and are further associated with higher cost. Additionally, MR cisternography is invasive and requires intrathecal injection [2, 4]. Using a combination of modalities (i.e., HRCT and MRI) has also shown to improve the accuracy [1, 2].

Despite the use of multiple imaging modalities preoperatively in our patient, only operative exploration with the use of intrathecal fluorescein positively located the fistula. Intrathecal fluorescein is suggested to be safe at diluted doses with the benefit of localizing defects [15, 16]. Intrathecal fluorescein is, however, invasive in nature, and some studies have failed to show a significant benefit [17].

The best localization technique would be one that is widely available, relatively low in cost, noninvasive, and highly accurate. This case highlights the fact that such a technique does not currently exist. Also, a thorough literature search reveals that most published reports on CSF leak identification are retrospective series with relatively small patient populations. Additional research is needed to determine the best approach to CSF leak localization.

After a thorough literature review, we identified 3 case reports of post-ACFD fistula presenting as a neck mass and dysphagia [7–9] and one case of spinal hardware eroding into the esophagus resulting in CSF leak and pneumocephalus [10]. These cases were uncommon, but even these all occurred within one month of surgery. The case we present is interesting because of its location in the oropharynx, but even more so due to the delayed nature. This highlights that CSF leaks can occur even five years after ACDF.

## 4. Conclusion

CSF leak after ACDF is an uncommon complication in the postoperative period, and it is a rare fistula to develop years after the procedure.

Despite using several imaging modalities to identify the CSF leak location, none of these identified the site of fistula in our patient. This case highlights the need for research to identify the best approach for defect localization that is accurate, cost effective, and minimally invasive.

## References

[1] Shetty P. G., Shroff M. M., Sahani D. V., Kirtane M. V. (1998). Evaluation of high-resolution CT and MR cisternography in the diagnosis of cerebrospinal fluid fistula. *American Journal of Neuroradiology*.

[2] Oakley G. M., Alt J. A., Schlosser R. J., Harvey R. J., Orlandi R. R. (2015). Diagnosis of cerebrospinal fluid rhinorrhea: an evidence-based review with recommendations. *International Forum of Allergy and Rhinology*.

[3] Zuckerman J. D., DelGaudio J. M. (2008). Utility of preoperative high-resolution CT and intraoperative image guidance in identification of cerebrospinal fluid leaks for endoscopic repair. *American Journal of Rhinology*.

[4] Ohwaki K., Yano E., Shinohara T. (2014). Spinal cerebrospinal fluid leaks detected by radionuclide cisternography and magnetic resonance imaging in patients suspected of intracranial hypotension. *Advances in Medical Sciences*.

[5] Syre P., Bohman L.-E., Baltuch G., Le Roux P., Welch W. C. (2014). Cerebrospinal fluid leaks and their management after anterior cervical discectomy and fusion. *Spine*.

[6] Bertalnaffy H., Eggert H. R. (1989). Complications of anterior cervical spine discectomy without fusion in 450 consecutive patients. *Acta Neurochirurgica*.

[7] Schaberg M. R., Altman J. I., Shapshay S. M., Woo P. (2007). Cerebrospinal fluid leak after anterior cervical disc fusion: an unusual cause of dysphagia and neck mass. *Laryngoscope*.

[8] Fountas K. N., Kapsalaki E. Z., Johnston K. W. (2005). Cerebrospinal fluid fistula secondary to dural tear in anterior cervical discectomy and fusion. *Spine*.

[9] Nair S. B., Flood L. M., Nath F. (1998). An unusual complication of Cloward’s procedure presenting to the otolaryngologist. *Journal of Laryngology and Otology*.

[10] Goodwin R. C., Boone C. E., Pendleton J. (2016). Pneumocephalus leading to the diagnosis of cerebrospinal fluid leak and esophageal perforation after cervical spine surgery. *Journal of Clinical Neuroscience*.

[11] Quadri S. A., Capua J., Ramakrishnan V. (2017). A rare case of pharyngeal perforation and expectoration of an entire anterior cervical fixation construct. *Journal of Neurosurgery: Spine*.

[12] Kuo Y.-C., Levine M. S. (2010). Erosion of anterior cervical plate into pharynx with pharyngotracheal Fistula. *Dysphagia*.

[13] Martín-Martín C., Martínez-Capoccioni G., Serramito-García R., Espinosa-Restrepo F. (2012). Surgical challenge: endoscopic repair of cerebrospinal fluid leak. *BMC Research Notes*.

[14] Daele J., Goffart Y., Machiels S. (2011). Traumatic, iatrogenic, and spontaneous cerebrospinal fluid (CSF) leak: endoscopic repair. *B-ENT*.

[15] Keerl R., Weber R. K., Draf W., Wienke A., Schaefer S. D. (2004). Use of sodium fluorescein solution for detection of cerebrospinal fluid fistulas: an analysis of 420 administrations and reported complications in Europe and the United States. *Laryngoscope*.

[16] Moseley J. I., Carton C. A., Stern W. E. (1978). Spectrum of complications in the use of intrathecal fluorescein. *Journal of Neurosurgery*.

[17] Seth R., Rajasekaran K., Benninger M. S., Batra P. S. (2010). The utility of intrathecal fluorescein in cerebrospinal fluid leak repair. *Otolaryngology Head and Neck Surgery*.

